# The (r)evolution of hyped innovations in orthopedic implants: can prudent introduction avoid throwing the baby out with the bathwater?

**DOI:** 10.1080/17453674.2019.1669115

**Published:** 2019-09-25

**Authors:** Job L C van Susante

**Affiliations:** Department of Orthopedics, Rijnstate Ziekenhuis, Arnhem, The Netherlands

Multiple new orthopedic implant designs are introduced into the market yearly. The satisfying results already achieved with most available implants challenge efforts to create additional value without introducing new patient risks. In weighing the balance between stimulating the evolution and improvement of musculoskeletal implants/medical devices and avoiding the introduction of potential risks, innovations should be introduced in a regulated, safe manner to prevent harm to patients. In spite of a consensus framework (IDEAL), which recommends the phased introduction of new medical devices (McCulloch et al. 2009), this introductory process still appears to be quite complex.

Recent innovations in orthopedic devices have often been embraced by both professionals and patients after popularity has increased from industrial marketing, which in turn has led to rapidly increasing clinical use. This phenomenon of the “Hype Cycle” is well known from consumer markets and may also well be applicable to illustrate the evolution of healthcare-related innovations (Bortfeld and Marks 2013). In this model, product evolution is divided into 3 phases (Figure 1). First, the skeptical resistance phase goes from novel technology invention towards early clinical use. Second, in the hype phase the new technology is subject to optimistic mass consumer uptake to a peak of inflated expectations; the transition from skeptical resistance to hype often happens abruptly (potential benefits outweigh potential risks). Third, the post-hype phase is when users gain a more realistic understanding of the strengths and weaknesses of the new technology. In fact, the post-hype phase is most interesting since it determines whether a sustainable plateau of productivity is achieved for an innovation or whether disillusion leads to stagnation or even obsolescence. Medical innovations are particularly vulnerable to a rapid drop into obsolescence in the post-hype phase since they are subject to multiple post-market surveillance and regulations because patients’ health is involved. As such, a hyped (market and technology driven) introduction of an orthopedic innovation may not only put patients at risk but may also negatively affect sustainable productivity of the innovation itself, ultimately increasing risks of eventually “throwing out the baby with the bath water.”

**Figure 1.**
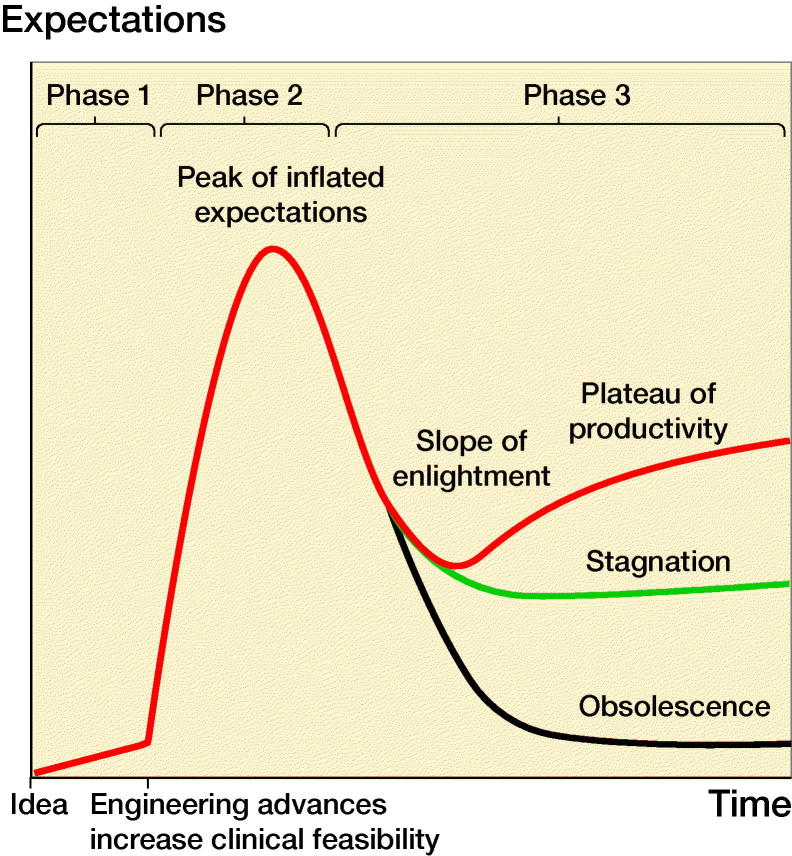
The 3 phases of the “Hype Cycle”: 1. Skeptical resistance phase; 2. Hype phase; 3. Post-hype phase (modified from Bortfeld and Marks 2013).

The awareness that a hyped introduction in itself may have a negative effect on the evolution towards clinical use of an innovation may be at least as effective to avoid such “hype” as the introduction of all sorts of regulations by notified bodies. This cultural change may then improve patient safety whilst in the meantime not frustrating innovations. Lessons can be learnt from examples of the introductory process of orthopedic innovations in the past. Analysis of the number of yearly publications may reflect whether the respective innovations underwent a hyped introduction or not (phase 2) and whether this predisposed towards a plateau of productivity in phase 3 or towards obsolescence. Several recent examples can illustrate the case.

## I. Modular necks for THA

**Figure 2.**
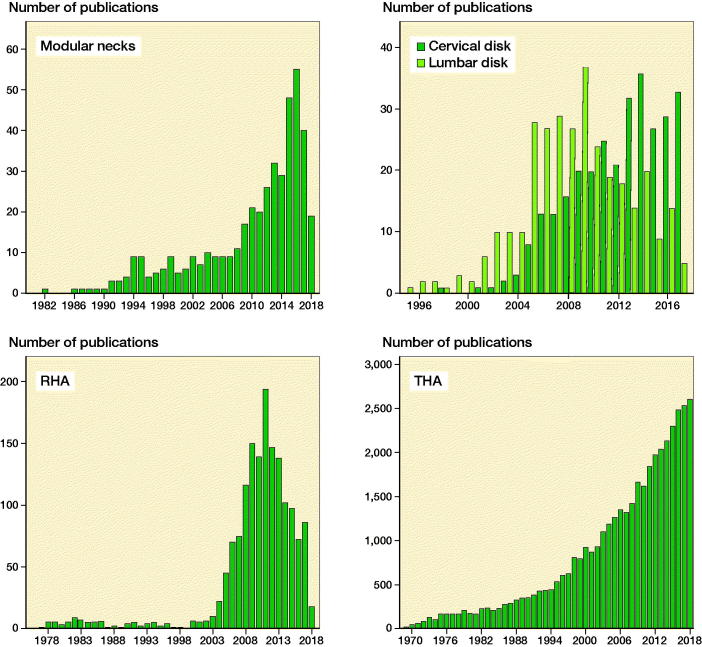
Annual number of publications registered in PubMed for 4 orthopedic innovations. The curves seem to overlap with the course of introduction in the market. 1.Modular necks in THA. A peak of inflated expectations is visible in 2016 after a “hyped” introduction, followed by a steep decline as concerns about safety appeared. Obsolescence in primary THA is likely to occur. 2.Resurfacing hip arthroplasty (RHA). A similar peak is visible in 2012 after a rapid uptake in the market, followed by a gradual decline from concerns around metal-on-metal issues. Stagnation occurs and obsolescence may be inevitable. 3.Total disc replacement (TDR). The use of lumbar disc arthroplasty increased rapidly after a “hyped” introduction in early 2000, followed by a steep decline towards obsolescence as long-term safety issues outweighed potential benefits. However, for cervical disc arthroplasty a gradual increase in evidence and acceptance in clinical use can be observed. 4.Total hip arthroplasty (THA). There has been no “hype phase” and an annually increasing number of publications corresponds with the established position in the market and a growing plateau of productivity.

Recurrent dislocation is one of the most important reasons for early revision of THA (Gerhardt et al. 2014). Modular necks were introduced in THA to improve restoration of hip geometry and reduce dislocation rates. Modular necks enhanced the opportunities for the surgeon to personalize anteversion, retroversion, varus and valgus orientation of the stem intraoperatively. Initially this innovation was reserved for revision stem designs and from the preliminary results encountered the concept was expanded towards the much larger market of primary THA. Strong industry marketing strategies resulted in rapid market uptake and a typical hype phase was initiated. Subsequently, inflated expectations of reduced dislocation rates were not met, and instead concerns were raised about adverse reaction to metal debris (ARMD) from corrosion, third body wear at the taper junction and occasional implant fracture (Vundelinckx et al. 2013). The trend in annual publications on PubMed concerning taper modularity in primary THA typically followed the hyped introduction of this innovation and uptake into the market (Figure 2). The peak of inflated expectations appears to have been reached in 2016, followed by a rapid decline, associated with concerns regarding patient safety. Since the originally presumed benefits for patients did not seem to hold (Gerhardt et al. 2014) the use of modular necks in primary THA appears to reach obsolescence in phase 3 of the hype cycle.

## II. Resurfacing hip arthroplasty (RHA)

This was designed for young and active patients and was reintroduced as a revolutionizing bone-preserving concept decades after resistance to an earlier introduction in the 1950s (phase 1). Following improved metallurgy in metal-on-metal (MoM) bearing, a hyped introduction followed from several different manufacturers (phase 2). At the same time the number of annual publications rapidly increased in the years 2006 to 2011 (Figure 2). Disillusion with the product (phase 3) was marked by reports of unexpectedly high metal ion release and high early revision rates (Smith et al. 2012). Subsequently, a rapid drop in annual publications coincided with a steep decline in clinical use from 2012 through 2018 (Figure 2). The RHA concept is at risk of reaching obsolescence in phase 3 of the hype cycle.

One could argue whether this is entirely justified. In the disillusion phase concerns around MoM bearings were obtained from combined data of the RHA and large-head MoM THA. Only later did it appear that bearing issues had to be distinguished from trunnion issues in MoM THA and that the latter were clearly more disturbing. As such, MoM THA may have taken down the RHA in its fall, whereas there may still be a minor niche for RHA. In specialized high-volume centers excellent 10-year survival rates have been reported; however, real-world data from different joint registries still report relatively high revision rates for RHA. From randomized controlled trials comparing RHA with conventional THA we have learnt that, besides somewhat more natural weightbearing in gait analysis (Gerhardt et al. 2019), no clear benefit could be detected in clinical outcome for RHA. Concerns around the MoM bearing remain eminent and as such the potential for significant patient risks from metal-on-metal issues so far outweigh the established minimal potential benefits over conventional THA.

## III. Total disc replacement (TDR)

Total vertebral disc replacement was introduced as an innovative implant design to treat degenerative disc disease. The theoretical advantage of TDR over spinal fusion is that movement is preserved and that, as such, adjacent segment degeneration could be avoided or minimized. Again, this innovation was targeted for young and active patients. From the year 2000 both cervical and lumbar disc arthroplasties gained popularity followed by a rapid uptake in the market. Similar to the situation with modular necks and RHA a “hype cycle” could be observed where a steep increase in clinical use paralleled increasing numbers of annual publications (Figure 2). Subsequently, meta-analyses comparing both lumbar and cervical TDR with fusion (Ding et al. 2017, Findlay et al. 2018, Katsuura et al. 2019) reported at least equivalent clinical outcome for TDR in the short term. Some subtle benefits seemed to apply more to cervical TDR, but were not beyond the generally accepted clinical important differences. Concerns were also expressed that disadvantages may appear after years as uncertainty remains about degeneration of the prosthesis (Jacobs et al. 2013).

Gradually inflated expectations subsided and in particular for lumbar disc arthroplasty the annual number of surgeries decreased dramatically from 3,059 to 420 from 2005 towards 2013 in the United States (Saifi et al. 2018a). Again, this decrease in clinical use coincided with a steep decline in the number of publications (Figure 2).

Interestingly, for cervical disc replacement a different pattern can be described with, as opposed to the lumbar disc replacement, a slope of enlightenment in phase 3 towards a plateau of productivity in clinical practice (Figure 2). Contrary to lumbar TDR, the annual number of cervical TDRs increased 190% from 540 to 1,565 from 2006 towards 2013 in the United States (Saifi et al. 2018b). The annual number of publications followed this pattern or vice versa. The rationale behind this different pattern is difficult to understand from similar conclusions in meta-analyses for both cervical and lumbar TDR meta-analysis. Fortunately the increase in clinical use of cervical TDR is still monitored by an increase in scientific evidence, which will determine whether the potential clinical benefit will eventually outweigh the risk for (long term) complications and revision need.

## IV. Total hip arthroplasty (THA)

The evolution of the THA, introduced as an innovation in orthopedics by Sir John Charnley (Charnley 1961), shows a distinct pattern. The innovation has been carefully incorporated into the market represented by gradually increasing numbers of implantations with careful monitoring of results. There was no “hype cycle” and subsequently the acceptance of the innovations could be monitored by a stepwise increase in the annual number of publications (Figure 2).

### Hype or (r)evolution

In general there is a tendency to overestimate the benefit of an innovation in the short run and underestimate the potential new risks in the long run. From the examples presented we have learnt that the curve of the annual number of publications of an innovation correlates with the evolution of its use. A “hyped” introduction may predispose to failure to meet with inflated expectations, followed by a steep decline in usage and subsequent banning of the innovation.

Besides patient safety concerns, these findings suggest that it may also benefit the innovation itself and the manufacturers’ interest in implementing a stepwise introduction and avoiding hype. Hype may actually predispose to obsolescence. Several regulatory measures have already been taken to improve monitoring of the introduction of future orthopedic innovations (Howard 2016). However, regulatory changes only will probably lead to suffocation of innovation, slowing the progress of healthcare innovation and improvement, and potentially slowing value creation for society. Ideally, the first step with prudent introduction of innovations would be careful monitoring of a limited number of patients treated. Only after clinical success has been warranted at least at short-term follow-up, without the introduction of new complications, could subsequent expansion of clinical use be pursued. A randomized controlled trial (RCT) where the innovation is benchmarked against the “standard of care” remains the ideal choice to evaluate the true clinical value of an innovation. Since conventional RCTs are elaborate, expensive, and commonly limited in potential patient recruitment, registry-nested RCTs are likely to gain a more dominant role. Typically, in these trials a novel approach or implant is compared with the standard of care technique in a pragmatic multicenter setting where the study is incorporated in daily clinical practice and clinically relevant outcome parameters can be obtained from available national registry data. For example, the potential benefit of decreased dislocation rates of a dual mobility cup over conventional cups is currently evaluated in such way in the “Duality” and the “Redep” trial (clinicaltrials.gov NCT03909815 and NCT04031820, respectively). Besides, a cultural change is necessary to help stakeholders understand that a “hyped introduction” of innovations should be avoided at all times. A cultural shift is advocated from sales- or fashion-driven short-term attention towards sustainability of health in long-term gain in health and quality of life (Porter 2010). From the annual publication reports of recent innovations we have learnt that a rapid uptake of a new device in the market most likely predisposes to subsequent stagnation or even obsolescence in phase 3 as inflated expectations are not met or new risks appear. For this reason, marketing of an innovation by companies should focus on building scientific evidence and as such preserving the sustainability of a new device. Professionals in turn will have to embrace this approach and avoid rapid uptake of devices without solid evidence; the time of a “boys need toys” approach is over. And, importantly, patients will have to understand that “new is not allows better.”

Parts of this manuscript were taken from the PhD thesis “Innovative Implant Design in Hip Arthroplasty” at the Radboud University Medical Centre, Nijmegen (Netherlands) by Davey M Gerhardt under co-supervision with Marinus de Kleuver (Professor and Chair of the Department of Orthopedics). Data from the annual publication graph on TDR were kindly provided by Richard D Guyer, MD, Center for Disc Replacement at the Texas Back Institute.

Job L C VAN SUSANTE *Department of Orthopedics, Rijnstate Ziekenhuis, Arnhem, The Netherlands* *Correspondence:* jvansusante@rijnstate.nl

